# Longitudinal Evaluation of the Effect of Tricyclic Antidepressants and Neuroleptics on the Course of Huntington’s Disease—Data from a Real World Cohort

**DOI:** 10.3390/brainsci11040413

**Published:** 2021-03-25

**Authors:** Jannis Achenbach, Carsten Saft, Simon Faissner

**Affiliations:** Huntington Center North Rhine-Westphalia, Department of Neurology, Ruhr-University Bochum, St. Josef-Hospital Bochum, Gudrunstraße 56, 44791 Bochum, Germany; carsten.saft@rub.de (C.S.); simon.faissner@rub.de (S.F.)

**Keywords:** Huntington’s disease, neuroprotection, tricyclic antidepressants, neuroleptics, ENROLL-HD

## Abstract

**Background:** Reducing the progress of neurodegeneration is a key goal in Huntington´s disease (HD). A previously performed systematic screening for medications with neuroprotective features identified tricyclic antidepressants and neuroleptics as neuroprotective and mitochondrioprotective agents. Here, we analyzed the characteristics of disease manifestation, progression and potential beneficial effects in HD patients treated with afore-mentioned medications compared to un- and otherwise treated motor-manifest patients in a large real-world cohort over two years. **Methods:** We analyzed cross-sectional data of the largest cohort worldwide of motor-manifest HD patients using the ENROLL-HD database, including demographic, moleculargenetic, clinical-motoric, cognitive and functional data. Longitudinal data of up to two years were obtained to analyze potential effects on disease progression between groups with different medications used. Data were analyzed using repeated ANOVA-analyses while controlling for the co-variates age and CAG-repeat length. **Results:** We identified *n* = 7397 motor-manifest HD patients using no or different medication (HD-ctrl) and subgroups treated with clomipramine (*n* = 56), clozapine (*n* = 66), chlorpromazine (*n* = 17), doxepine (*n* = 34) and desi-, imi- or trimipramine (*n* = 19). Demographic parameters, disease onset and CAP-score did not differ. Total motor scores (TMS) at baseline were higher in patients treated with clozapine (*p* < 0.001), chlorpromazine and clomipramine (*p* < 0.05) compared to HD-ctrl with higher sub scores for bradykinesia (all *p* < 0.01) and dystonia in clozapine treated patients (*p* < 0.001). Functional and cognitive capacities were worse in medication groups in comparison to HD-ctrl at baseline (*p* < 0.001). Repeated measures analysis of variance documented no differences regarding motoric, functional and cognitive disease progressions between groups. **Conclusions:** We identified group differences, potentially caused by side effects or potential selection bias in terms of bradykinetic motoric symptoms, more dystonia and lower functional and cognitive performance in some treatment groups at baseline, which were not entirely explained because of underlying fundamental characteristics. Disease progression regarding clinical, functional and cognitive outcomes over two years was not affected by any of the treatment groups compared to HD-ctrl. Our data do not support our hypothesis of a potential neuroprotective effect of these drugs on disease progression.

## 1. Introduction

The autosomal-dominant neurodegenerative disorder Huntington’s disease (HD) is characterized by the occurrence of heterogeneous motoric, cognitive and psychiatric symptoms [1,2]. During the last decade, several pivotal research advances have been made concerning the understanding of the pathobiology and molecular-genetic causes of HD, leading to innovative and potential beneficial therapeutic options which are currently in the developmental pipeline [3]. Manifold therapeutic approaches are in the preclinical but also clinical development process, primarily aiming to lower the mutant huntingtin protein with gene-silencing approaches such as antisense oligonucleotides, so-called “small molecules” influencing pre-mRNA splicing mechanisms, RNA interference targeting mRNA, zinc finger transcriptional repressors or CRISPR-Cas 9 approaches [4]. Another approach targeting somatic expansion are antisense oligonucleotides or “small molecules”, potentially inducing a contraction of the expanded CAG in the brain [5,6,7]. Until now, however, no finally proven beneficial neuroprotective therapy is available [8].

A crucial pillar of HD treatment is the use of neuroleptics used for symptomatic treatment of diverse HD symptoms. Beneficial effects of neuroleptics are known for the treatment of typical symptoms such as chorea. However, the only US Food and Drug Administration-approved drug for treatment of chorea in HD is currently tetrabenazine and deutetrabenazine [9]. The situation is challenging for the treatment of other hyperkinetic movement-disorders such as dystonia or myoclonus with only case report series published for treatment recommendations [10,11,12] or predominant bradykinetic phenotypes especially in children [13,14]. Psychiatric symptoms such as depression and anxiety also lack standardized treatment strategies [15], often based on case reports or survey-based expert opinions with the aim to optimize individual therapeutic approaches [16,17,18]. For the management of neuropsychiatric symptoms as well as motoric and functional disability the use of the atypical neuroleptic clozapine has been described as a strategy with partly beneficial symptomatic effects [19,20,21,22].

Depression occurs in HD with high prevalence, and therefore several pharmacological interventions can be implemented using selective serotonin reuptake inhibitors, multiple-receptor antidepressants and tricyclic antidepressants as partly effective symptomatic approaches [23,24]. The tricyclic antidepressant doxepine is used as first-line medication for insomnia in older adults [25,26,27] as well as to treat depression and anxiety [28,29,30]. Information concerning the evaluating of the use and indication of doxepine in patients suffering from HD are missing until now. Other pharmaceuticals allocated to the group of tricyclic antidepressants are desi-, imi- and trimipramine used in patients with major depression, primary focusing, next to clinical aspects, on pharmacokinetic and side effects [31,32,33,34,35]. Trimipramine has additionally been used in patients with mixed symptoms accompanied by depression, anxiety and insomnia [36,37]. Imipramine is used in obsessive-behavior, chronic pain and enuresis [38,39]. Especially potentially anticholinergic side-effects with worsening of cognition upon use of tricyclic antidepressants might limit their use in HD [40]. Until now, however, knowledge is scarce regarding the use of tricyclic antidepressants in HD. 

The antipsychotic chlorpromazine is, independent of HD, commonly used for treatment of psychoses and especially for schizophrenia with considerable side effects [41]. Chlorpromazine is, independent from HD, commonly used for treatment of psychosis and especially for schizophrenia with considerable side effects such as sedation and parkinsonism.

Since causative treatment approaches, described above, are still lacking, adapting already approved and tested generic available medications might, at this juncture, be essential for the treatment of the complex HD pattern. We previously performed a screening to identify neuroprotective orally available generic medications with known neuroprotective features against iron mediated neurotoxicity, a pathomechanism relevant for progressive multiple sclerosis [42]. Here, we could identify a group of neuroprotective antidepressants such as clomipramine as well as antipsychotics such as clozapine, which moreover also elicited mitochondrioprotective properties [42], a pathomechanism also relevant in HD [1,43]. Antidepressants have positive effects on various cellular neurodegenerative mechanisms with a great neuroprotective potential [44]. Promising neuroprotective effects also have been shown for desipramine and imipramine in preclinical models of HD, describing desipramine and imipramine as candidates for beneficial effects on mitochondrial protection [45]. Imipramine additionally revealed positive effects for memory preservation as well as on motor improvement and desipramine showed positive effects against apoptosis in mouse-models of HD [45].

In this study, we evaluated how antidepressants and antipsychotics are used in HD. Moreover, we investigated the question of whether those medications, used in HD as symptomatic therapy, might have an effect on the progression of HD regarding motor, functional and cognitive outcomes both cross-sectionally and longitudinally over two years. To address this question, we took advantage of the ENROLL-HD database, the largest observational study in HD worldwide [46].

## 2. Methods

### 2.1. ENROLL-HD Database and Patients

We analyzed motor-manifest HD participants of the global cohort of the ENROLL-HD-study. Enroll-HD is a global clinical research platform designed to facilitate clinical research in HD. Core datasets are collected annually from all research participants as part of this global multi-center longitudinal observational study. Data are monitored for quality and accuracy using a risk-based monitoring approach. All sites are required to obtain and maintain local ethical approval. The periodic dataset four (PDS4) was investigated and inclusion-criteria were set concerning age (>18 years), a diagnostic confidence level (DCL) of 4 (having unequivocal signs of clinical manifest HD (>99% confidence), a total motor-score (TMS) >5 and a genetically confirmed report with ≥36 cytosine-adenine-guanine (CAG)-repeats in the Huntingtin gene (*HTT*). We identified motor-manifest participants treated with the tricyclic antidepressants clomipramine, chlorpromazine, doxepine and desi-, imi- and trimipramine or the antipsychotic clozapine (HD-treated). As an HD-control group (HD-ctrl) without afore-mentioned specific antidepressant or antipsychotic medication we identified *n* = 7397 participants meeting the inclusion criteria, who were either un- or otherwise treated in order to compare disease manifestation and progression. Frequently prescribed other drugs were: tetrabenazine (*n* = 1937), olanzapine (*n* = 1724), risperidone (*n* = 1026), haloperidol (*n* = 585), tiapride (*n* = 391), sulpiride (*n* = 337) and amantadine (*n* = 313). Using a cross-sectional approach we compared baseline data of study entry. Patients were identified using outlined medication starting before their initial visit, whereby demographical, motoric and clinical (with onsets of HD-motor and non-motor symptoms noticed by the participant, family, clinical rater and age at HD diagnosis), functional and cognitive parameters were assessed during baseline. In addition, follow-up data were obtained and analyzed in patients of whom annual data (±3 months) had been collected up to two more years. Additional moleculargenetic parameters were assessed by using CAG-repeat lengths and by calculating the CAG-Age Product-Index (CAP-score) [47]. Furthermore, indications for medication-intake were assessed. Motoric parameters of symptomatic disease manifestation were analyzed using the Unified Huntington’s Disease Rating Scale (UHDRS)-Total motor score (TMS) and additionally by calculating sub scores for chorea, hypokinetic-rigidity and dystonia [48,49,50].

Cognitive performance was analyzed using the ENROLL-HD test battery of five cognitive tests: Symbol digit modality test (SDMT), Verbal fluency test (Verfct), Stroop color naming (SCN), Stroop word reading (SWR) and Stroop interference test (SIT). Functionality was analyzed with the UHDRS-Total functional capacity (TFC) and Independence Scale (IS).

### 2.2. Statistical Analyses

**Figure 1:**
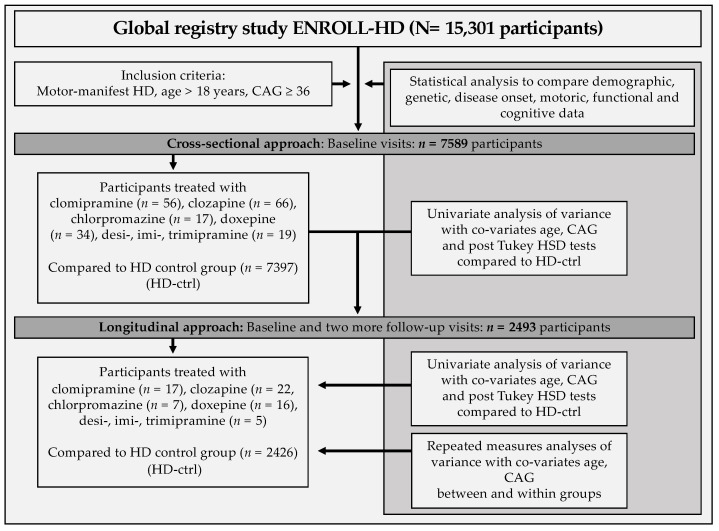
Schematic workflow of the study.

Group means and standard deviation were assessed using ANOVA-analysis controlling for Co-variates (age and CAG) during baseline-visit with post hoc Tukey HSD tests in IBM SPSS Statistics V.25. Afterwards, repeated measures ANOVA-analysis with co-variates were conducted to determine differences between medication-subgroups and non-medication group with longitudinal data. Additionally, repeated measures ANOVA-analyses were conducted within each specific group comparing their own group-performance between baseline and follow-up visit two (Figure 1).

## 3. Results

### 3.1. Age, Age at HD Diagnosis and CAP-Score Did Not Differ between Controls and HD-Treated Patients

We identified *n* = 7589 motor-manifest HD participants from the Enroll-HD periodic dataset four who met the inclusion criteria. Univariate analysis of variance with co-variates age and CAG were used. Patients were compared regarding their fundamental demographic characteristics and motoric, functional and cognitive disease manifestation during baseline visit. Subgroups treated with clomipramine (*n* = 56), clozapine (*n* = 66), chlorpromazine (*n* = 17), doxepine (*n* = 34) or desi-, imi- and trimipramine (*n* = 19) were identified.

In total, two participants in the clomipramine, one in the clozapine, none in the chlorpromazine, two in the doxepine and six in the desi-, imi- and trimipramine group had started medication-intake before symptoms of the motor-manifest HD disease had been present. The remainder of patients had started after the onset of motor-symptoms compatible with HD.

Demographic and fundamental disease parameters such as age of participants, age of HD diagnosis or symptom onset observed by the clinical rater did not differ between the groups (Table 1). We also calculated the CAP-score, which integrates CAG-repeat length and age, which also did not differ. The CAG-repeat length, however, depicted differences regarding the analysis of all groups (*p* < 0.029), while post hoc analyses did not differ. Of note, symptom onset observed by the patient and family also differed upon analysis of the whole group with significantly worse values in the clozapine group compared to HD-ctrl (Sxsubj *p* < 0.05; Sxfam *p* < 0.01).

### 3.2. HD Patients Treated with Antidepressants or Antipsychotics Had More Motor and Functional Impairment at Baseline

Having established that baseline criteria regarding age, age of HD diagnosis and symptom onset as well as CAP-score did not differ, we first wanted to analyze whether motor symptoms assessed using the UHDRS-total motor score differed at baseline. Interestingly, patients treated with clozapine (*p* < 0.001), clomipramine (*p* < 0.05) and chlorpromazine (*p* <0.01) had significantly higher TMS compared to HD-ctrl. The doxepine and desi-, imi-, trimipramine group did not differ compared to HD-ctrl. We further investigated sub scores for chorea, dystonia and bradykinesia within the UHDRS-TMS-scores, which depicted that patients treated with clozapine were more dystonic and bradykinetic affected compared to HD-ctrl (*p* < 0.001). In addition, bradykinetic symptoms were more present in patients treated with clomipramine (*p* < 0.01) and chlorpromazine (*p* < 0.01) compared to HD-ctrl. The other groups did not differ. 

We then set out to investigate differences in functional outcomes and analyzed functional assessments in the UHDRS-Total functional capacity score and Independence scale. We found that patients in the clomipramine, clozapine and chlorpromazine group performed worse in all tests during the baseline visit compared to HD-ctrl participants (all *p* < 0.001), with the lowest functional activity in patients treated with chlorpromazine. Analyses of cognitive capacities revealed similar differences with lower performance in the clomipramine, clozapine and chlorpromazine groups compared to HD-ctrl (all *p* < 0.001) using a battery of five cognitive tests. In analogy to the findings of functional activity, the chlorpromazine group revealed the worst cognitive performance in all tests at baseline (Table 1). 

We analyzed indications for the administered medication in the respective groups (Supplementary Table S1). The indication for the use of clomipramine was in most cases depression (*n* = 32), followed by obsessive-compulsive disorders (*n* = 11) and anxiety (*n* = 7). Clozapine was administered mostly because of a psychotic disorder (*n* = 34), delusion (*n* = 6) and schizophrenia (*n* = 4). Chlorpromazine-treated patients suffered from psychotic disorder (*n* = 7), not closer named abnormal behavior (*n* = 2) and irritability (*n* = 2). Doxepine was mostly administered as antidepressant (*n* = 26) and because of a present sleep disorder with implemented insomnia (*n* = 11). The indication for the use of desi-, imi- and trimipramine was similar to the doxepine group due to depression and depressed mood (*n* = 13) as well as a sleep disorder with insomnia (*n* = 7) (Supplementary Table S1).

### 3.3. Similar Motor, Functional and Cognitive Decline in a Longitudinal Analysis of Two Years 

To analyze whether treatment with aforementioned medications might influence the HD course, we analyzed longitudinal data of up to two years follow-up. We identified *n* = 2426 HD-ctrl patients with follow-up data for two more follow-up visits (annual interval ± 3 months) and *n* = 17 patients in the clomipramine, *n* = 22 in the clozapine, *n* = 7 in the chlorpromazine, *n* = 16 in the doxepine and *n* = 5 in the desi-, imi-, trimipramine group. Similar to the baseline situation, demographic and fundamental disease parameters did not differ (Table 2). 

Similar to the baseline situation which included the analysis of all patients, we found that in patients of whom follow-up data of two years existed the TMS was significantly higher in patients treated with clozapine (*p* < 0.001) and chlorpromazine (*p* < 0.01) compared to the HD-ctrl group (Table 2). The chorea sub score did not differ. Patients treated with clozapine, however, suffered significantly more from dystonia and bradykinesia compared to HD-ctrl (*p* < 0.001). Moreover, patients treated with both clozapine (*p* < 0.001) and chlorpromazine (*p* < 0.01) had significant high-grade bradykinesia compared to HD-ctrl.

We further assessed whether functional and cognitive parameters differed at baseline in this longitudinal cohort. Indeed, functional and cognitive assessments differed significantly in all tests (all *p* < 0.05). Patients treated with clozapine and chlorpromazine had significantly worse baseline data than HD-ctrl. Average results for all cognitive and functional tests were lowest for the chlorpromazine group. Patients treated with clomipramine, doxepine or desi-, imi- and trimipramine did not show significant group differences in pair-wised post hoc tests compared to HD-ctrl during their baseline visit (Table 2).

We then set out to investigate whether one of the baseline parameters might differ in follow-up analyses depending on the medication taken. We therefore performed repeated measures analyses of variance with co-variates age and CAG and analyzed motoric, functional and cognitive capacities. As expected, we could observe a gradual decline in all assessed parameters.

**Figure 2:**
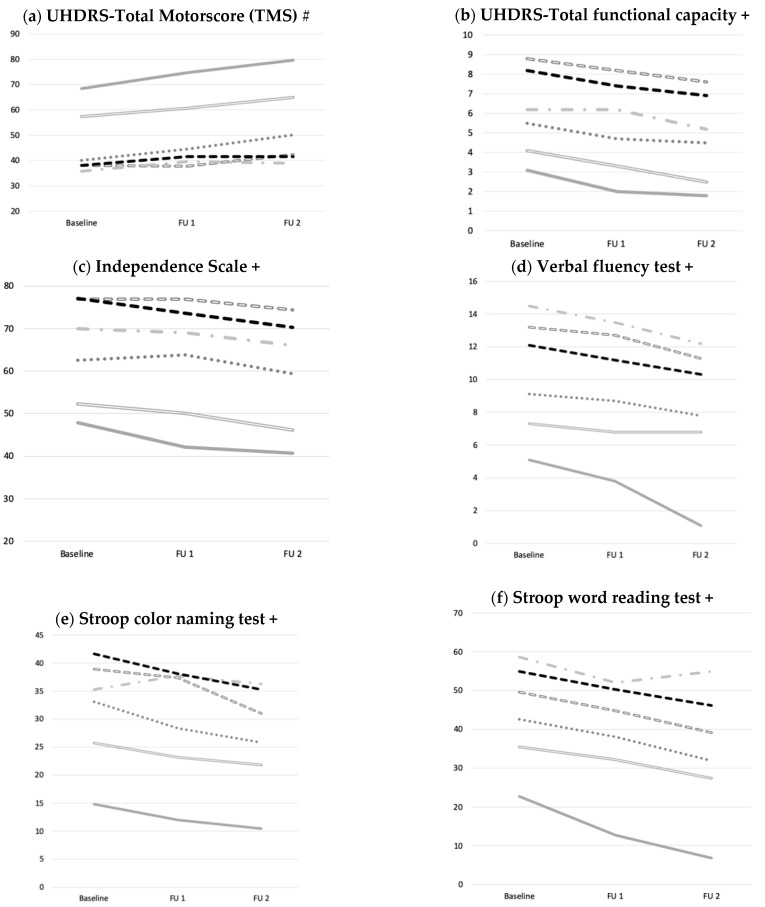

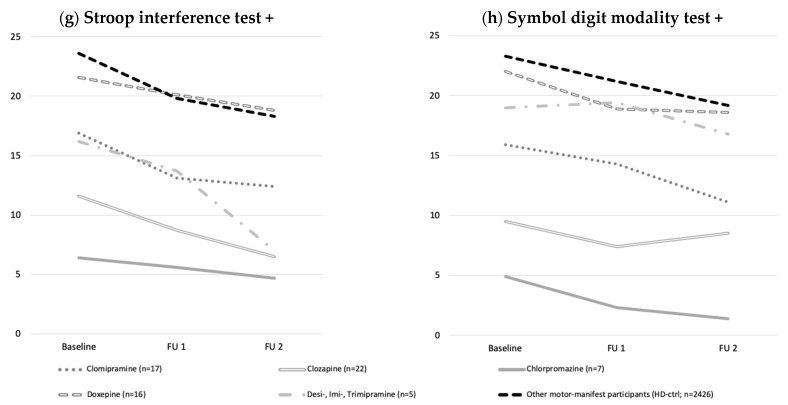
Development of motoric, functional and cognitive performance over two years. (**a**) Motor (UHDRS TMS), (**b**,**c**) functional, (**d**–**h**) and cognitive performance declined over time without effect of medication taken compared to HD-ctrl. Data were analyzed using ANOVAs with co-variates (age, CAG) for baseline, follow up one and two, comparing respective medication- and HD-ctrl over time. Abbreviations: UHDRS: Unified Huntington’s Disease Rating Scale; FU1: Follow up one; FU2: Follow up two; #: higher scores= more impairment; +: higher scores= better performance.

There was, however, no effect on the decline depending on the medication taken compared to HD-ctrl (Table 3; Figure 2). The TMS in the HD-ctrl group increased from 38.1 ± 20.0 (mean ± SD) to 45.4 ± 21.8 after two years.

To assess performance within the medication groups and within the control group, longitudinal data between the respective baseline and follow up visit two were analyzed in each group. Repeated measures analyses of variance considering motoric, functional and cognitive performance revealed significant decreases in all tests in the clomipramine, clozapine, doxepine and HD-ctrl group (all *p* < 0.001). Participants treated with chlorpromazine (*n* = 7) significantly declined regarding the motoric symptoms and regarding functionality within the Independence Scale (all *p* < 0.005) as well as in the Verbal fluency test (*p* = 0.048) and Stroop word reading test (*p* = 0.045). Patients treated with desi-, imi- and trimipramine (*n* = 5) declined in all aspects apart from the SDMT (Table 3 and Table 4).

## 4. Discussion

Although causative treatment approaches for HD are underway, it might still take time until those therapeutics will be available in daily clinical practice. Therefore, reducing symptom severity and the velocity of the decline of a plethora of neurological domains still remains a key goal of current therapeutic approaches. We here set out to investigate for the first-time effects of several tricyclic antidepressants and the antipsychotic clozapine taking advantage of the worldwide largest cohort of HD patients using the ENROLL-HD database.

We identified a group of 7397 motor-manifest HD patients without specific treatment and subgroups of patients which were treated with aforementioned medications. Baseline criteria regarding demographic parameters and age at HD diagnosis did not differ between medication subgroups and HD-ctrl. While the CAG differed in the analysis of all groups, there were no specific group differences in post hoc analyses. Remarkably, we did not observe any differences concerning the CAP-score, which is an index used to quantify disease progression or the also called “toxic-load” as key marker variable [47]. Thus, fundamental group variables of the medication- and control groups were mostly comparable, therefore excluding an influence of heterogeneous fundamental moleculargenetic, demographic or “pathobiological” aspects on cognitive, functional or motor- disease manifestation [51,52,53]; a strength of the data presented here.

Motor aspects did, however, differ between respective groups at baseline, with clozapine, chlorpromazine and clomipramine treated patients being more impaired than the control group regarding the TMS. To better understand which motor aspect was most affected, we also calculated sub scores for chorea, hypokinesia and dystonia. Taking those data into account, we were able to show that TMS differences were mostly related to bradykinesia in patients treated with clomipramine or chlorpromazine as well as more dystonic symptoms in patients treated with clozapine. This might be related to the anticholinergic side-effect profile related to clomipramine or the iatrogenic parkinsonism related to chlorpromazine treatment [54,55]. Clozapine, on the contrary, is only rarely used in HD due to its side effects and the need for repeated blood tests; it might have been used especially in bradykinetic or dystonic but psychotic HD patients, hence a distinct subgroup of patients. Moreover, the subject and family estimate of symptom onset was significantly worse in this group at baseline, while the clinical diagnosis did not differ, indicating that subtle symptoms might have been apparent earlier, potentially explaining a selection bias accounting for the group differences observed in clozapine treated patients. This is supported by the indications documented for the use of clozapine in our patients.

Respective medication groups did not only perform worse regarding motor aspects, but were also significantly more impaired regarding functional and cognitive domains compared to HD-ctrl. This might be related to sedative effects, known to negatively impact on cognition and functionality in patients treated with neuroleptics and tricyclic antidepressants [56]. This, however, remains to be proven since sedative side-effects of other neuroleptics or antidepressants in the comparison group cannot be excluded. Those effects were stable over time with clozapine and chlorpromazine treated patients being more dystonic and bradykinetic and less cognitive and functional active. Stability over time supports the idea that those effects might be side-effects and not be due to disease progression.

The reasons for prescriptions between the different groups differed substantially; medications were mostly started due to psychiatric symptoms, as expected. The antidepressant clomipramine, for example, is usually prescribed in patients with obsessive-compulsive disorders; this now can be confirmed with regard to the use in HD, which was not investigated until now to the best of our knowledge [57,58]. Next to obsessive-compulsive behavior, thoughts and irritability (*n* = 17), clomipramine was prescribed for depression (*n* = 32), anxiety (*n* = 7) and in one case to treat perseverations, which are commonly known in HD as marker for poor cognitive flexibility and difficult to treat [59,60].

The neuroleptic clozapine is usually used to treat psychiatric disorders in HD [19], a finding confirmed in our study. Apart from the potential sedative side-effects of both clozapine and chlorpromazine, psychiatric disorders might have been potential factors negatively impacting on cognition and functional capacities; a finding, having been described in patients suffering from schizophrenia [61]. Depression was the main indication for doxepine and desi-, imi- and trimipramine treatment. In addition, the sleep-inducing effect of doxepine was also used in some of our patients as the second most important reason for prescription. There were manifold further indications in the desi-, imi- and trimipramine group such as enuresis in one case, having been described independently from HD [39].

Apart from cross-sectional data in this large HD cohort, we analyzed longitudinal data to better understand dynamics of motor, functional and cognitive decline depending on presumed neuroprotective comedications—a huge advantage of our study. This is certainly of interest, since until now there remains a considerable knowledge gap regarding the effects of aforementioned medications on long-term outcomes and disease progression in HD. We had longitudinal data of a considerable number of patients with n = 2426 in the HD-ctrl group. Remarkably, we did not observe faster decline regarding motoric, functional and cognitive disease progression in any of the groups analyzed. This was an unexpected finding both from daily clinical experience as well as in light of the baseline data with considerably higher impairment in the medication groups, suggesting that those patients might progress faster. One possible explanation might be that baseline differences might have been due to side-effects or selection bias, as discussed earlier, without affecting disease progression negatively. Since it would not have been surprising if treated patients would have progressed faster, since those were already more severely affected at baseline, it might be suspected that the medications used might have induced a stabilizing effect. Although a clear and distinctive neuroprotective effect on disease progression in any of the medication groups is not evident, a stabilizing effect can be hypothesized and would be one explanation for the unaltered decline over two years. This, however, remains highly suggestive and cannot be proven at this point. The side effects thereby did not seem to have influenced further disease progression negatively if compared to progression in HD-ctrl patients without specific medication.

Limitations of the study presented here are the relatively small sample size in the treatment groups and the retrospective design. Moreover, the use of other CNS affecting medications in the HD-ctrl group such as antidepressants with differing mechanisms, e.g., SSRI, might have been a confounding factor in our control group, which could not be ruled out as potential bias. The strengths of the data presented here consist of the large cohort, recruited at multiple sites world-wide and the involvement of longitudinal data over two years.

## 5. Conclusions

In summary, we identified group differences, potentially caused by side effects or selection bias in terms of bradykinetic motoric symptoms, more dystonia and lower functional and cognitive performance in some treatment groups at baseline, which were not entirely explained by underlying fundamental characteristics. All aspects investigated, namely motor function, functionality, and cognitive function, declined over time in all groups without differences between the groups. We did neither observe clear beneficial nor detrimental effects of named antidepressants or antipsychotics longitudinally. We therefore conclude that the medications investigated in this study seem to not have any detrimental effect on the clinical course of HD patients over a two-year period in this real-world setting. Our data do not support our hypothesis of a potential neuroprotective effect of these drugs on disease progression.

## Figures and Tables

**Table 1:** Baseline data of HD-ctrl and treated HD patients.

	Clomipramine *n* = 56	Clozapine n = 66	Chlorpromazine *n* = 17	Doxepine *n*= 34	Desi-, Imi-, Trimipramine *n* = 19	Other Motor-Manifest(HD-ctrl) *n* = 7397	F	*p*	Part. Eta^2^
Age (y); M (SD) (Co-variate CAG)	52.7 (12.3)	50.6 (12.4)	49.9 (15.6)	55.1 (11.2)	53.6 (13.3)	53.2 (12.6)	0.446	0.817	0.000
CAG high (Co-variate age)	45.3 (6.0)	45.0 (3.8)	46.3 (6.1)	43.4 (3.1)	44.5 (5.0)	44.1 (4.0)	2.491	0.029	0.002
CAP-score (no Co-variate)	555.6 (109.5)	539.8 (108.2)	557.4 (113.1)	513.0 (100.9)	534.0 (122.9)	540.3 (213.8)	0.197	0.964	0.000
Hddiagn (y)	44.9 (13.3)	44.5 (12.9)	42.7 (15.2)	49.9 (10.1)	47.6 (13.7)	48.6 (13.1)	0.580	0.715	0.000
Sxrater (y)	41.7 (12.8)	38.5 (12.2)	37.8 (14.9)	44.2 (13.3)	40.2 (11.4)	42.4 (16.3)	0.329	0.895	0.000
Sxsubj (y)	41.6 (12.9)	40.1 (12.8) *	42.0 (15.6)	46.7 (11.9)	43.3 (13.4)	45.9 (13.3)	2.429	0.033	0.002
Sxfam (y)	40.4 (13.7)	38.7 (12.6) **	38.4 (15.4)	46.6 (10.3)	41.3 (12.9)	45.2 (13.3)	3.549	0.005	0.003
Motoric UHDRS TMS; M (SD) #	51.5 (23.4) *	59.0 (22.7) ***	61.4 (25.3) **	45.1 (25.6)	47.9 (28.0)	40.0 (21.5)	17.085	<0.001	0.011
Sub score Chorea; M (SD) #	6.7 (4.3)	7.2 (5.7)	6.9 (6.2)	8.4 (5.0)	6.6 (3.9)	8.0 (4.6)	1.81	0.107	0.001
Sub score Dystonia; M (SD) #	4.6 (5.1)	5.8 (5.3) ***	6.9 (5.9)	4.4 (5.5)	6.9 (6.6)	3.4 (4.3)	9.19	<0.001	0.006
Sub score Hypo-Bradykinesia; M (SD) #	13.7 (6.9) **	14.9 (6.5) ***	15.8 (6.8) **	10.9 (6.8)	12.8 (8.1)	10.0 (6.0)	16.29	< 0.001	0.011
TFC; M (SD) +	5.0 (3.4) ***	3.8 (3.0) ***	3.4 (2.8) ***	7.3 (3.8)	6.1 (3.9)	7.9 (3.7)	27.872	<0.001	0.018
IS; M (SD) +	58.6 (21.3) ***	52.4 (19.5) ***	48.2 (22.1) ***	71.2 (18.7)	68.1 (22.9)	75.3 (19.1)	34.572	<0.001	0.022
SDMT; M (SD) +	11.6 (12.7) ***	7.3 (9.4) ***	6.0 (7.8) ***	19.4 (12.9)	17.5 (15.8)	22.0 (12.7)	30.612	<0.001	0.022
Verfct; M (SD) +	8.0 (6.6) ***	6.6 (5.3) ***	6.2 (5.0) ***	11.3 (6.3)	11.5 (7.5)	11.6 (5.8)	17.119	<0.001	0.012
SCNT; M (SD) +	26.3 (18.8) ***	20.9 (16.7) ***	17.9 (15.2) ***	34.5 (17.6)	35.5 (19.3)	40.3 (17.7)	28.493	<0.001	0.019
SWRT; M (SD) +	34.4 (24.7) ***	28.0 (21.8) ***	27.8 (19.3) ***	46.3 (24.0)	46.3 (24.6)	53.6 (23.5)	27.185	<0.001	0.019
SIT; M (SD) +	11.2 (11.4) ***	8.6 (11.7) ***	6.3 (9.1) ***	18.5 (11.5)	15.5 (13.4)	22.7 (11.5)	41.043	<0.001	0.032

Cross-sectional univariate analysis of variance (Co-Variates: CAG, age) with baseline-data of all analyzed participants. Post hoc Tukey HSD tests with medication groups versus other motor-manifest participants (significance testing: * = *p* < 0.05; ** = *p* < 0.01; *** = *p* < 0.001). Abbreviations: M: mean; SD: standard deviation; *p*: *p* value; F: F value; Part Eta^2^: Effect size; UHDRS: Unified Huntington’s Disease Rating Scale; CAG: cytosine-adenine-guanine repeat length; CAP-score: CAG-Age Product- Index; Hddiagn: Huntington´s disease diagnosed; Sxrater: Rater’s estimate of symptom onset; Sxsubj: Subject estimate of symptom onset; Sxfam: Families estimate of symptom onset; TMS: Total motor score; TFC: Total functional capacity; IS: Independence scale; SDMT: Symbol digit modality test; Verfct: Verbal fluency test; SCNT: Stroop color naming test; SWRT: Stroop word reading test; SIT: Stroop interference test; Mg: milligram; Y: years; #: higher scores= more impairment; +: higher scores= better performance.

**Table 2:** Baseline data of patients with two years follow-up.

	Clomipramine *n* = 17	Clozapine *n* = 22	Chlorpromazine *n* = 7	Doxepine *n* = 16	Desi-, Imi-, Trimipramine *n* = 5	Other Motor-Manifest (HD-ctrl) *n* = 2426	F	*p*	Part. Eta^2^
Age (y); M (SD) (Co-variate CAG)	56.1 (10.3)	51.4 (12.0)	50.6 (17.7)	52.2 (9.5)	52.0 (8.7)	53.1 (12.1)	0.407	0.844	0.001
CAG high (Co-variate age)	43.3 (1.7)	44.3 (3.6)	47.0 (7.6)	43.1 (3.1)	42.8 (2.3)	44.0 (3.9)	1.16	0.299	0.002
CAP-score (no Co-Variate)	528.1 (63.1)	514.8 (115.2)	573.5 (81.4)	471.7 (74.1)	479.6 (84.3)	547.1 (254.1)	0.484	0.788	0.001
Hddiagn (y)	49.8 (8.6)	46.3 (11.4)	43.8 (14.7)	48.7 (8.8)	48.0 (8.3)	48.4 (12.4)	0.542	0.744	0.001
Sxrater (y)	45.9 (8.4)	38.9 (10.9)	39.0 (13.7)	44.4 (10.3)	45.2 (7.9)	43.0 (14.8)	0.398	0.851	0.001
Sxsubj (y)	46.7 (8.5)	41.9 (11.7)	40.8 (17.0)	44.5 (9.9)	44.8 (8.2)	45.7 (12.5)	0.934	0.458	0.002
Sxfam (y)	47.2 (8.9)	39.2 (10.4)	40.0 (15.3)	46.8 (8.1)	45.6 (7.9)	44.9 (12.6)	1.544	0.173	0.003
Motoric UHDRS TMS; M (SD) #	40.1 (18.6)	57.4 (26.2) ***	68.4 (30.0) **	38.1 (20.0)	35.8 (23.4)	38.1 (20.0)	7.215	<0.001	0.014
Sub score Chorea; M (SD) #	6.8 (3.2)	9.2 (5.4)	8.6 (8.0)	8.1 (5.2)	5.5 (5.0)	7.9 (4.6)	1.020	0.404	0.002
Sub score Dystonia; M (SD) #	2.1 (3.6)	6.6 (6.0) ***	7.9 (6.9)	3.2 (3.9)	6.8 (6.0)	3.1 (4.1)	6.571	<0.001	0.011
Sub score Hypo-Bradykinesia; M (SD) #	10.1 (6.1)	15.0 (6.5) ***	15.4 (7.9) **	9.0 (4.9)	9.8 (5.6)	9.3 (5.6)	6.848	<0.001	0.012
TFC; M (SD) +	5.5 (3.1)	4.1 (3.0) ***	3.1 (2.9) ***	8.8 (3.0)	6.2 (3.1)	8.2 (3.5)	11.909	<0.001	0.023
IS; M (SD) +	62.6 (20.4)	52.3 (18.7) ***	47.8 (23.8) ***	76.9 (9.5)	70.0 (14.6)	77.1 (17.3)	15.686	<0.001	0.030
SDMT; M (SD) +	15.9 (14.6)	9.5 (12.3) ***	4.9 (7.2) **	22.0 (12.0)	19.0 (18.3)	23.3 (12.4)	9.580	<0.001	0.020
Verfct; M (SD) +	9.1 (6.6)	7.3 (5.3) *	5.1 (5.7) **	13.2 (5.2)	14.5 (10.4)	12.1 (5.7)	6.103	<0.001	0.012
SCNT; M (SD) +	33.1 (16.8)	25.7 (17.7) ***	14.8 (20.0) **	38.9 (13.3)	35.3 (22.9)	41.7 (17.3)	7.953	<0.001	0.016
SWRT; M (SD) +	42.6 (23.4)	35.4 (23.5) **	22.7 (23.3) **	49.6 (18.0)	58.7 (19.8)	55.0 (22.6)	7.139	<0.001	0.015
SIT; M (SD) +	16.9 (10.7)	11.6 (13.9) ***	6.4 (11.0) **	21.6 (11.4)	16.2 (7.1)	23.6 (11.4)	10.175	<0.001	0.023

Univariate Analysis of Variance (Co-Variates: CAG, age) of baseline-data of patients with two more follow-up visits in a cross-sectional approach. Post hoc Tukey HSD tests comparing respective medication groups versus other motor-manifest participants (HD-ctrl). Significance is depicted as: * = *p* < 0.05; ** = *p* < 0.01; *** = *p* < 0.001. Abbreviations: M: mean; SD: standard deviation; p: p value; F: F value; Part Eta^2^: effect size; UHDRS: Unified Huntington’s Disease Rating Scale; CAG: cytosine-adenine-guanine repeat length; CAP-score: CAG-Age Product- Index; Hddiagn: Huntington´s disease diagnosed; Sxrater: Rater’s estimate of symptom onset; Sxsubj: Subject estimate of symptom onset; Sxfam: Families estimate of symptom onset; TMS: Total motor score; TFC: Total functional capacity; IS: Independence scale; SDMT: Symbol digit modality test; Verfct: Verbal fluency test; SCNT: Stroop color naming test; SWRT: Stroop word reading test; SIT: Stroop interference test; Mg: milligram; Y: years; #: higher scores= more impairment; +: higher scores = better performance.

**Table 3:** Longitudinal analyses of motor, functional and cognitive parameters.

	Clomipramine *n* = 17			Clozapine *n* = 22			Chlorpromazine *n* = 7			Doxepine *n* = 16			Desi-, Imi-, Trimipramine *n* = 5			Other Motor-Manifest (HD-ctrl) *n* = 2426			F	*p*	Part. Eta^2^
	BL	FU 1	FU 2	BL	FU1	FU2	BL	FU1	FU2	BL	FU1	FU2	BL	FU1	FU2	BL	FU1	FU2			
UHDRS TMS; M (SD) #	40.1 (18.6)	44.5 (18.5)	50.1 (21.7)	57.4 (26.2)	60.7 (21.9)	64.9 (23.5)	68.4 (30.0)	74.7 (30.0)	79.7 (29.5)	38.1 (20.0)	37.7 (19.8)	42.3 (20.2)	35.8 (23.4)	39.6 (25.6)	39.0 (23.8)	38.1 (20.0)	41.5 (20.8)	45.4 (21.8)	0.818	0.603	0.002
TFC; M (SD) +	5.5 (3.1)	4.7 (3.4)	4.5 (3.7)	4.1 (3.0)	3.3 (2.6)	2.5 (2.6)	3.1 (2.9)	2.0 (1.8)	1.8 (2.9)	8.8 (3.0)	8.2 (2.8)	7.6 (2.4)	6.2 (3.1)	6.2 (3.1)	5.2 (3.3)	8.2 (3.5)	7.4 (3.5)	6.9 (3.6)	0.452	0.906	0.001
IS; M (SD) +	62.6 (20.4)	63.8 (19.2)	59.4 (19.0)	52.3 (18.7)	50.0 (17.7)	46.1 (20.4)	47.8 (23.8)	42.1 (21.2)	40.7 (20.9)	76.9 (9.5)	76.9 (11.1)	74.4 (12.1)	70.0 (14.6)	69.0 (14.7)	66.0 (15.6)	77.1 (17.3)	73.6 (18.0)	70.3 (18.9)	0.872	0.559	0.002
SDMT; M (SD) +	15.9 (14.6)	14.3 (14.2)	11.1 (12.6)	9.5 (12.3)	7.4 (12.1)	8.5 (12.4)	4.9 (7.2)	2.3 (6.0)	1.4 (3.7)	22.0 (12.0)	18.9 (9.5)	18.6 (13.6)	19.0 (18.3)	19.4 (20.5)	16.8 (21.4)	23.3 (12.4)	21.2 (12.8)	19.2 (13.5)	0.966	0.470	0.002
Verfct; M (SD) +	9.1 (6.6)	8.7 (6.5)	7.8 (7.6)	7.3 (5.3)	6.8 (5.2)	6.8 (5.5)	5.1 (5.7)	3.8 (4.4)	1.1 (2.0)	13.2 (5.2)	12.7 (3.2)	11.3 (5.5.)	14.5 (10.4)	13.5 (11.7)	12.2 (8.1)	12.1 (5.7)	11.2 (6.0)	10.3 (6.0)	0.089	1.00	0.000
SCNT; M (SD) +	33.1 (16.8)	28.4 (16.1)	25.8 (18.1)	25.7 (17.7)	23.2 (17.5)	21.8 (17.6)	14.8 (20.0)	12.0 (18.1)	10.4 (19.0)	38.9 (13.3)	37.4 (11.6)	31.0 (18.4)	35.3 (22.9)	37.7 (20.0)	36.3 (22.1)	41.7 (17.3)	38.1 (18.3)	35.3 (18.7)	0.132	0.997	0.000
SWRT; M (SD) +	42.6 (23.4)	38.1 (24.3)	31.9 (24.5)	35.4 (23.5)	32.1 (23.6)	27.3 (23.7)	22.7 (23.3)	12.7 (17.3)	6.7 (13.2)	49.6 (18.0)	44.8 (19.1)	39.1 (24.6)	58.7 (19.8)	52.0 (23.4)	55.0 (21.8)	55.0 (22.6)	50.3 (24.5)	46.1 (24.8)	0.176	0.993	0.000
SIT; M (SD) +	16.9 (10.7)	13.1 (12.3)	12.4 (11.3)	11.6 (13.9)	8.7 (12.4)	6.5 (12.1)	6.4 (11.0)	5.6 (10.9)	4.7 (12.5)	21.6 (11.4)	20.1 (9.3)	18.8 (14.2)	16.2 (7.1)	13.7 (10.7)	7.0 (9.2)	23.6 (11.4)	19.8 (13.2)	18.3 (13.1)	0.333	0.953	0.001

Gradual decline in all groups with no significant alteration depending on the medication taken compared to HD-ctrl. Data are depicted as group- means (standard deviation). Data were analyzed using repeated measures analysis of variance with Co-Variates (age, CAG) for baseline, follow up one and two. Abbreviations: M: mean; SD: standard deviation; P: p value; F: F value; Part Eta^2^: effect size; UHDRS: Unified Huntington’s Disease Rating Scale; TMS: Total motor score; TFC: Total functional capacity; IS: Independence scale; SDMT: Symbol digit modality test; Verfct: Verbal fluency test; SCNT: Stoop color naming test; SWRT: Stroop word reading test; SIT: Stroop interference test; #: higher scores= more impairment; +: higher scores= better performance.

**Table 4:** Comparison of baseline analysis and follow-up after two years.

	Clomipramine *n* = 17			Clozapine *n* = 22			Chlorpromazine *n* = 7			Doxepine *n* = 16			Desi-, Imi-, Trimipramine *n* = 5			Other Motor-Manifest *n* = 2426		
	F	*p*	Part. Eta^2^	F	*p*	Part. Eta^2^	F	*p*	Part. Eta^2^	F	*p*	Part. Eta^2^	F	*p*	Part. Eta^2^	F	*p*	Part. Eta^2^
TMS; M (SD) #	88.515	<0.001	0.847	144.904	<0.001	0.873	44.407	<0.005	0.881	79.357	<0.001	0.841	12.632	0.024	0.759	10,436.269	<0.001	0.811
TFC; M (SD) +	43.175	<0.001	0.120	34.423	<0.001	0.621	87.500	0.051	0.496	160.535	<0.001	0.915	16.327	0.016	0.803	12,194.549	<0.001	0.833
IS; M (SD) +	171.688	<0.001	0.121	151.568	<0.001	0.878	28.687	<0.005	0.827	868.043	<0.001	0.983	109.769	<0.001	0.965	43,953.511	<0.001	0.948
SDMT; M (SD) +	17.695	<0.005	0.525	71.247	<0.001	0.772	2.977	0.135	0.332	41.774	<0.001	0.736	4.111	0.136	0.578	68.67644	<0.001	0.750
Verfct; M (SD) +	25.706	<0.001	0.616	43.012	<0.001	0.672	6.159	0.048	0.507	92.703	<0.001	0.861	8.138	0.046	0.670	12,311.611	<0.001	0.838
SCNT; M (SD) +	51.934	<0.001	0.764	42.516	<0.001	0.669	4.613	0.075	0.435	87.817	<0.001	0.854	18.678	0.012	0.824	14580.643	<0.001	0.861
SWRT; M (SD) +	17.096	<0.001	0.517	40.450	<0.001	0.658	6.413	0.045	0.517	82.828	<0.001	0.847	32.884	0.005	0.892	14333.078	<0.001	0.859
SIT; M (SD) +	35.880	<0.001	0.692	10.742	<0.001	0.338	1.621	0.250	0.213	42.678	<0.001	0.740	21.598	0.010	0.844	3706.899	<0.001	0.797

Data were analyzed using repeated measures analysis of variance within groups between baseline and follow up two. Mean data are depicted in Table 3. Abbreviations: M: mean; SD: standard deviation; P: p value; F: F value; Part Eta^2^: effect size; TMS: Total motor score; TFC: Total functional capacity; IS: Independence scale; SDMT: Symbol digit modality test; Verfct: Verbal fluency test; SCNT: Stoop color naming test; SWRT: Stroop word reading test; SIT: Stroop interference test; #: higher scores= more impairment; +: higher scores= better performance.

## Data Availability

The data that support the findings of this study are available from the corresponding author upon reasonable request.

## Supplementary Materials

The following are available online at https://www.mdpi.com/2076-3425/11/4/413/s1, Table S1: Treatment indications. Indication for analyzed medications separated depending on respective groups during baseline visit.

## References

[1] Walker F.O. (2007). Huntington’s disease. Lancet.

[2] Young A.B., Shoulson I., Penney J.B., Starosta-Rubinstein S., Gomez F., Travers H., Ramos-Arroyo M.A., Snodgrass S.R., Bonilla E., Moreno H. (1986). Huntington’s disease in Venezuela: Neurologic features and functional decline. Neurology.

[3] Tabrizi S.J., Flower M.D., Ross C.A., Wild E.J. (2020). Huntington disease: New insights into molecular pathogenesis and therapeutic opportunities. Nat. Rev. Neurol..

[4] Wild E.J., Tabrizi S.J. (2017). Therapies targeting DNA and RNA in Huntington’s disease. Lancet Neurol..

[5] Flower M.D., Tabrizi S.J. (2020). A small molecule kicks repeat expansion into reverse. Nat. Genet..

[6] Nakamori M., Panigrahi G.B., Lanni S., Gall-Duncan T., Hayakawa H., Tanaka H., Luo J., Otabe T., Li J., Sakata A. (2020). A slipped-CAG DNA-binding small molecule induces trinucleotide-repeat contractions in vivo. Nat. Genet..

[7] Therapeutics, Inc. Preventing Repeat Expansion Disorders at Their Source. https://www.triplettx.com/approach/Triplet.

[8] Dickey A.S., La Spada A.R. (2018). Therapy development in Huntington disease: From current strategies to emerging opportunities. Am. J. Med. Genet. Part A.

[9] Richard A., Frank S. (2019). Deutetrabenazine in the treatment of Huntington’s disease. Neurodegener. Dis. Manag..

[10] Shannon K.M., Fraint A. (2015). Therapeutic advances in Huntington’s Disease. Mov. Disord..

[11] Saft C., Lauter T., Kraus P.H., Przuntek H., Andrich J.E. (2006). Dose-dependent improvement of myoclonic hyperkinesia due to Valproic acid in eight Huntington’s Disease patients: A case series. BMC Neurol..

[12] Saft C., Von Hein S.M., Lucke T., Thiels C., Peball M., Djamshidian A., Heim B., Seppi K. (2018). Cannabinoids for Treatment of Dystonia in Huntington’s Disease. J. Huntington’s Dis..

[13] Scahill R.I., Zeun P., Osborne-Crowley K., Johnson E.B., Gregory S., Parker C., Lowe J., Nair A., O’Callaghan C., Langley C. (2020). Biological and clinical characteristics of gene carriers far from predicted onset in the Huntington’s disease Young Adult Study (HD-YAS): A cross-sectional analysis. Lancet Neurol..

[14] Achenbach J., Thiels C., Lücke T., Saft C. (2020). Clinical Manifestation of Juvenile and Pediatric HD Patients: A Retrospective Case Series. Brain Sci..

[15] Wyant K.J., Ridder A.J., Dayalu P. (2017). Huntington’s Disease—Update on Treatments. Curr. Neurol. Neurosci. Rep..

[16] Kane J.M., Leucht S., Carpenter D., Docherty J.P. (2003). The expert consensus guideline series. Optimizing pharmacologic treatment of psychotic disorders. Introduction: Methods, commentary, and summary. J. Clin. Psychiatry.

[17] Anderson K.E., Van Duijn E., Craufurd D., Drazinic C., Edmondson M., Goodman N., Van Kammen D., Loy C., Priller J., Goodman L.V. (2018). Clinical Management of Neuropsychiatric Symptoms of Huntington Disease: Expert-Based Consensus Guidelines on Agitation, Anxiety, Apathy, Psychosis and Sleep Disorders. J. Huntington’s Dis..

[18] Bachoud-Lévi A.-C., Ferreira J., Massart R., Youssov K., Rosser A., Busse M., Craufurd D., Reilmann R., De Michele G., Rae D. (2019). International Guidelines for the Treatment of Huntington’s Disease. Front. Neurol..

[19] Sharma M., Aggarwal S., Ragavi N., Kumar M. (2020). Management of neuropsychiatric symptoms in Huntington’s disease (HD) with clozapine: A case report. Asian J. Psychiatry.

[20] Van Vugt J.P.P., Siesling S., Vergeer M., Van Der Velde E.A., Roos R.A.C. (1997). Clozapine versus placebo in Huntington’s disease: A double blind randomised comparative study. J. Neurol. Neurosurg. Psychiatry.

[21] Colosimo C., Cassetta E., Bentivoglio A.R., Albanese A. (1995). Clozapine in Huntington’s disease. Neurology.

[22] Bonelli R., Wenning R.M.B.A.G.K. (2006). Pharmacological Management of Huntingtons Disease: An Evidence- Based Review. Curr. Pharm. Des..

[23] Slaughter J.R., Martens M.P., Slaughter K.A. (2001). Depression and Huntington’s Disease: Prevalence, Clinical Manifestations, Etiology, and Treatment. CNS Spectr..

[24] Ford M.F. (1986). Treatment of Depression in Huntington’s Disease with Monoamine Oxidase Inhibitors. Br. J. Psychiatry.

[25] Matheson E., Hainer B.L. (2017). Insomnia: Pharmacologic Therapy. Am. Fam. Phys..

[26] Edmonds C., Swanoski M., Chelsey E., Michael S. (2017). A Review of Suvorexant, Doxepin, Ramelteon, and Tasimelteon for the Treatment of Insomnia in Geriatric Patients. Consult. Pharm..

[27] Yeung W.-F., Chung K.-F., Yung K.-P., Ng T.H.-Y. (2015). Doxepin for insomnia: A systematic review of randomized placebo-controlled trials. Sleep Med. Rev..

[28] Ciurezu T., Timofte G. (1974). Doxepine therapy in outpatients with depressive anxious states. Rev. Roum. Med..

[29] van Renynghe d.V. (1972). La doxepine dans la dépression. Acta Psychiatr. Belg..

[30] Agabio R., Trogu E., Pani P.P. (2018). Antidepressants for the treatment of people with co-occurring depression and alcohol dependence. Cochrane Database Syst. Rev..

[31] De La Barquera J.A.O.-S. (2017). Respuesta a antidepresivos serotoninérgicos y noradrenérgicos: Estudio cruzado con fluoxetina y desipramina en pacientes con un primer episodio depresivo mayor. Gac. Med. Mex.

[32] Sallee F.R., Pollock B.G. (1990). Clinical Pharmacokinetics of Imipramine and Desipramine. Clin. Pharmacokinet..

[33] Nelson J.C. (1984). Use of desipramine in depressed inpatients. J. Clin. Psychiatry.

[34] Janowsky D.S., Byerley B. (1984). Desipramine: An overview. J. Clin. Psychiatry.

[35] Werry J.S. (1994). The Safety of Desipramine. J. Am. Acad. Child Adolesc. Psychiatry.

[36] Pecknold J.C., Luthe L. (1989). Trimipramine, anxiety, depression and sleep. Drugs.

[37] National Institute of Diabetes and Digestive and Kidney Diseases (2012). LiverTox: Clinical and Research Information on Drug-Induced Liver Injury. Trimipramine.

[38] Glassman A.H., Perel J.M. (1973). The Clinical Pharmacology of Imipramine. Arch. Gen. Psychiatry.

[39] Dinello F.A., Champelli J. (1968). The Use of Imipramine in the Treatment of Enuresis a Review of the Literature. Can. Psychiatr. Assoc. J..

[40] Richardson K., Fox C., Maidment I., Steel N., Loke Y.K., Arthur A., Myint P.K., Grossi C.M., Mattishent K., Bennett K. (2018). Anticholinergic drugs and risk of dementia: Case-control study. BMJ.

[41] Adams C.E., Awad G.A., Rathbone J., Thornley B., Soares-Weiser K. (2014). Chlorpromazine versus placebo for schizophrenia. Cochrane Database Syst. Rev..

[42] Faissner S., Mishra M., Kaushik D.K., Wang J., Fan Y., Silva C., Rauw G., Metz L., Koch M., Yong V.W. (2017). Systematic screening of generic drugs for progressive multiple sclerosis identifies clomipramine as a promising therapeutic. Nat. Commun..

[43] Saft C., Zange J., Andrich J., Müller K., Lindenberg K., Landwehrmeyer B., Vorgerd M., Kraus P.H., Przuntek H., Schöls L. (2005). Mitochondrial impairment in patients and asymptomatic mutation carriers of Huntington’s disease. Mov. Disord..

[44] Jamwal S., Kumar P. (2015). Antidepressants for neuroprotection in Huntington’s disease: A review. Eur. J. Pharmacol..

[45] Lauterbach E.C. (2013). Neuroprotective Effects of Psychotropic Drugs in Huntington’s Disease. Int. J. Mol. Sci..

[46] Landwehrmeyer G.B., Fitzer-Attas C.J., Giuliano J.D., Gonçalves N., Anderson K.E., Cardoso F., Ferreira J.J., Mestre T.A., Stout J.C., Sampaio C. (2016). Data Analytics from Enroll-HD, a Global Clinical Research Platform for Huntington’s Disease. Mov. Disord. Clin. Pr..

[47] Zhang Y., Long J.D., Mills J.A., Warner J.H., Lu W., Paulsen J.S., The PREDICT-HD Investigators and Coordinators of the Huntington Study Group (2011). Indexing disease progression at study entry with individuals at-risk for Huntington disease. Am. J. Med. Genet. Part B Neuropsychiatr. Genet..

[48] Kieburtz K., Penney J.B., Corno P., Ranen N., Shoulson I., Feigin A., Abwender D., Greenarnyre J.T., Higgins D., Marshall F.J. (1996). Unified Huntington’s disease rating scale: Reliability and consistency. Mov. Disord..

[49] Hart E.P., Marinus J., Burgunder J.-M., Bentivoglio A.R., Craufurd D., Reilmann R., Saft C., Roos R.A., the REGISTRY investigators of the European Huntington’s Disease Network (2013). Better global and cognitive functioning in choreatic versus hypokinetic-rigid Huntington’s disease. Mov. Disord..

[50] Achenbach J., Von Hein S.M., Saft C. (2020). Functional and cognitive capacity differ in dystonic motor subtypes when compared to choreatic and hypokinetic-rigid motor subtypes in Huntington’s disease. Brain Behav..

[51] Henley S.M.D., Wild E.J., Hobbs N.Z., Scahill R.I., Ridgway G.R., MacManus D.G., Barker R.A., Fox N.C., Tabrizi S.J. (2009). Relationship between CAG repeat length and brain volume in premanifest and early Huntington’s disease. J. Neurol..

[52] Wanker E.E., Ast A., Schindler F., Trepte P., Schnoegl S. (2019). The pathobiology of perturbed mutant huntingtin protein–protein interactions in Huntington’s disease. J. Neurochem..

[53] Sun Y.-M., Zhang Y.-B., Wu Z.-Y. (2016). Huntington’s Disease: Relationship Between Phenotype and Genotype. Mol. Neurobiol..

[54] Iversen T.S.J., Steen N.E., Dieset I., Hope S., Mørch R., Gardsjord E.S., Jørgensen K.N., Melle I., Andreassen O.A., Molden E. (2018). Side effect burden of antipsychotic drugs in real life—Impact of gender and polypharmacy. Prog. Neuro-Psychopharmacol. Biol. Psychiatry.

[55] Feinberg M. (1991). Clomipramine for obsessive-compulsive disorder. Am. Fam. Phys..

[56] Shoulson I. (1981). Huntington disease: Functional Capacities in patients treated with neuroleptic and antidepressant drugs. Neurology.

[57] Kelly M.W., Myers C.W. (1990). Clomipramine: A Tricyclic Antidepressant Effective in Obsessive Compulsive Disorder. DICP.

[58] Hoffmann R., Schröder N., Brüne M., Von Hein S., Saft C. (2019). Obsessive-Compulsive Symptoms are Less Common in Huntington’s Disease than Reported Earlier. J. Huntington’s Dis..

[59] De Lucia N., Peluso S., Roca A., De Michele G., Trojano L., Salvatore E. (2019). Perseverative Behavior on Verbal Fluency Task in Patients with Huntington’s Disease: A Retrospective Study on a Large Patient Sample. Arch. Clin. Neuropsychol..

[60] Oosterloo M., Craufurd D., Nijsten H., Van Duijn E. (2019). Obsessive-Compulsive and Perseverative Behaviors in Huntington’s Disease. J. Huntington’s Dis..

[61] Sheffield J.M., Barch D.M. (2016). Cognition and resting-state functional connectivity in schizophrenia. Neurosci. Biobehav. Rev..

