# Supplementary material for: Longitudinal Evaluation of the Effect of Tricyclic Antidepressants and Neuroleptics on the Course of Huntington’s Disease—Data from a Real World Cohort
Source: Brain Sci. 2021 Mar 25;11(4):413. doi: 10.3390/brainsci11040413 (PMC8064332; doi:10.3390/brainsci11040413)
Supplement: Supplementary file 1 [file brainsci-11-00413-s001.pdf]

SUPPLEMENT

Supplementary Table S1

**Clomipramine**	**Clozapine**	**Chlorpromazine**	**Doxepine**	**Desi-, Imi-, Trimipramine**
Anxiety (n=7)	Abnormal behavior (n=3)	Abnormal behaviour (n=2)	Antidepressant therapy (n=1)	Depressed mood (n=1)
Depression (n=32)	Affective disorder (n=2)	Affective disorder (n=1)	Anxiety (n=1)	Depression(n=12)
Insomnia (n=3)	Aggression (n=1)	Eructation (n=1)	Depression (n=25)	Duodenal ulcer (n=1)
Irritability (n=4)	Agitation (n=2)	Insomnia (n=1)	Fibromyalgia (n=1)	Enuresis (n=1)
Obsessive thoughts (n=2)	Chorea (n=1)	Irritability (n=2)	Insomnia (n=8)	Insomnia (n=5)
Obsessive-compulsive disorder (n=11)	Delusion (n=6)	Mild mental retardation (n=1)	Psychogenic pain disorder (n=1)	Mental disorder (n=1)
Perseveration (n=1)	Delusion mixed (n=1)	Mood swings (n=1)	Sleep disorder (n=3)	Salivary hypersecretion (n=1)
Schizoaffective disorder (n=1)	Dyskinesia (n=1)	Psychotic disorder (n=8)		Sleep disorder (n=2)
	Dystonia (n=1)			
	Hallucination (n=2)			
	Insomnia (n=1)			
	Irritability (n=2)			
	Obsessive thoughts (n=2)			
	Paranoia (n=1)			
	Perseveration (n=1)			
	Psychotic disorder (n=34)			
	Restlessness (n=1)			
	Schizoaffective disorder (n=1)			
	Schizophrenia (n=4)			
	Schizophrenia, paranoid type (n=3)			
	Screaming (n=1)			

Supplementary Table S1: Treatment indications. Indication for analyzed medications separated depending on respective groups during baseline visit (double entries implemented).

Abbreviation: n: number.
